# West Nile Virus, Texas, USA, 2012

**DOI:** 10.3201/eid1911.130768

**Published:** 2013-11

**Authors:** Kristy O. Murray, Duke Ruktanonchai, Dawn Hesalroad, Eric Fonken, Melissa S. Nolan

**Affiliations:** Baylor College of Medicine, Houston, Texas, USA (K.O. Murray,, M.S. Nolan);; Texas Children’s Hospital, Houston (K.O. Murray, M.S. Nolan);; Texas Department of State Health Services, Austin, Texas, USA (D. Ruktanonchai, D. Hesalroad, E. Fonken)

**Keywords:** West Nile virus, epidemiology, outbreak, economic impact, attack rates, Texas, viruses, vector-borne infections

## Abstract

During the 2012 West Nile virus outbreak in Texas, USA, 1,868 cases were reported. Male patients, persons >65 years of age, and minorities were at highest risk for neuroinvasive disease. Fifty-three percent of counties reported a case; 48% of case-patients resided in 4 counties around Dallas/Fort Worth. The economic cost was >$47.6 million.

West Nile virus (WNV) first emerged in Texas, USA, in 2002 (*1*). Since then, the virus has become endemic, with ≈2,200 human cases reported in the state during 2002–2011 (*2*). In 2012, an unprecedented outbreak of WNV occurred in Texas; ≈1,900 cases were reported. The objective of this study was to understand the epidemiology of the 2012 WNV outbreak in Texas.

## The Study

WNV infection is a reportable condition in Texas, with clinical cases passively reported by physicians to the local health departments, which in turn report to Texas Department of State Health Services (TxDSHS). We examined surveillance data for all reported cases for which symptom onset occurred during the 2012 calendar year, and we used descriptive statistics to describe the clinical features and demographic characteristics of reported case-patients. We calculated attack rates by sex, age, and race/ethnicity and incidence rates by county using population estimates for 2012 (*3*). Odds ratios (ORs), 95% CIs, and p values were calculated to determine differences in demographic variables between severe disease (WNV neuroinvasive disease [WNND], which included encephalitis, meningoencephalitis, and meningitis) and less severe disease (uncomplicated WNV fever). Epi Info 7.0 software (Centers for Disease Control and Prevention, Atlanta, GA, USA) was used for all statistical calculations.

**Figure 1:**
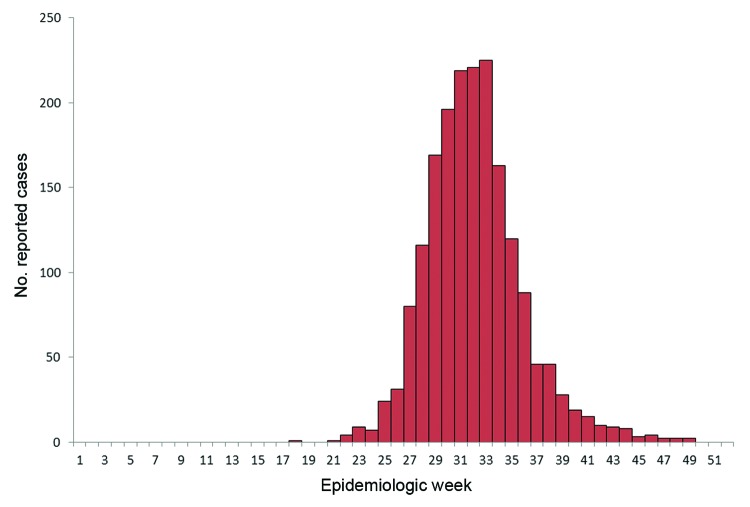
Number of reported West Nile virus cases, Texas, USA, 2012.

A total of 1,868 cases were reported to TxDSHS during the 2012 transmission season (Table), including 844 (45%) WNND cases and 89 deaths (case-fatality rate 5%). Dates of onset ranged from May 1, 2012, through December 6, 2012 (Figure 1). The outbreak peaked during week 33 (mid-August) with 225 reported cases, which is historically the same peak for all reported WNV cases in Texas during 2002–2011 (*2*). The median time from date of symptom onset to date of official report to TxDSHS was 27 days (range 6–274 days).

**Table:** Demographic characteristics and attack rates of all West Nile virus cases reported to the Texas (USA) Department of State Health Services during the 2012 outbreak

Characteristic	All cases, no. (%), n = 1,868	Attack rate*/100,000 population	WNV fever, no. (%), n = 1,024	WNV neuroinvasive disease, no. (%), n = 844	Deaths, no. (%), n = 89
Sex					
M	1,028 (55.0)	8.1	519 (50.7)	509 (60.3)	56 (62.9)
F	840 (45.0)	6.5	505 (49.3)	335 (39.7)	33 (37.1)
Age, y					
<18	70 (3.8)	1.0	42 (4.1)	28 (3.3)	0
18–24	71 (3.8)	2.7	42 (4.1)	29 (3.4)	0
25–44	439 (23.5)	6.2	283 (27.6)	156 (18.5)	5 (5.6)
45–64	728 (39.0)	11.7	424 (41.4)	304 (36.0)	13 (14.6)
>65	560 (30.0)	20.0	233 (22.8)	327 (38.7)	71 (79.8)
Race/ethnicity					
White, non-Hispanic	1,273 (68.1)	11.1	738 (72.1)	535 (63.4)	54 (60.7)
Black	117 (6.3)	4.0	43 (4.2)	74 (8.8)	1 (1.1)
White, Hispanic	318 (17.0)	3.2	134 (13.1)	184 (21.8)	22 (24.7)
Other/unknown	160 (8.6)	11.2	109 (10.6)	51 (6.0)	12 (13.5)

*Attack rates based on 2012 population estimates from the Texas State Data Center (*3*).

When examining the demographic characteristics of the reported cases, we found significant differences in sex, age, and race/ethnicity with regard to severity of disease. Overall, a higher percentage of male case-patients were reported (55%), and male case-patients were significantly more likely than female case-patients to have WNND (OR 1.5, 95% CI 1.2–1.8, p<0.001). Median age of all case-patients was 54 years (range 1 month–100 years). As each age category increased, the attack rates also increased (Table). Persons >65 years of age were significantly more likely than younger persons to have WNND (OR 2.1, 95% CI 1.8–2.6, p<0.001). The median age of the 89 case-patients who died was 79 years (range 25–100 years). When examining race/ethnicity of all cases, we observed the highest attack rate (11.1 cases/100,000 population) in white, non-Hispanics. However, minority populations were significantly more likely to have WNND (OR 1.9, 95% CI 1.6–2.4, p<0.001).

**Figure 2:**
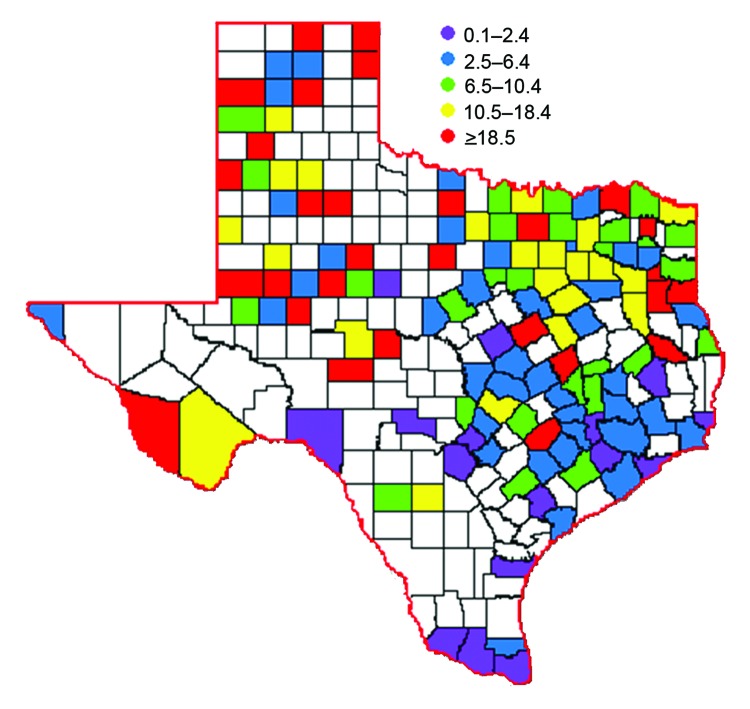
Incidence rates per 100,000 population during West Nile virus outbreak, Texas, USA, 2012. Numbers in parentheses indicate the number of counties that fell within each range.

Of the 254 counties in Texas, 135 (53%) reported a WNV case (Figure 2). The overall incidence rate for the state was 7.8 cases per 100,000 population. Almost half of the cases were reported from the northeastern quadrant of the state, including the Dallas/Fort Worth metroplex (902 [48%] cases): Dallas (396 [21%]), Tarrant (259 [14%]), Collin (64 [3%]), and Denton (183 [10%]) counties. These 4 counties had a combined incidence rate of 16 cases per 100,000 population.

## Conclusions

The 2012 WNV outbreak in Texas was unexpected in terms of the magnitude of virus transmission and number of human cases. We recently observed a 3-year pattern of increases in reported human cases in Texas, as seen in 2003, 2006, and 2009 (*2*). In 2012, the dramatic epidemic was consistent with this prior observation, with the 1,868 reported cases being more than double the historic high, which occurred in 2003 (735 cases). In addition to the dramatic increase in human cases in 2012, the state also reported an increase in equine cases (121 cases in 2012 compared with 6 cases in 2011). The exact factors that contributed to this epidemic are unknown and most likely complex, considering that successful transmission depends on supportive environmental conditions, vector abundance, avian reservoir and susceptible host abundance, pathogenicity of the virus, and sizeable populations of immunologically naive reservoir species.

WNV more severely affects persons >65 years of age; deaths typically are reported in elderly presons (*4*,*5*). During 2012, there was some media speculation that more cases of severe disease occurred in younger persons and that the circulating strain of virus possibly was more pathogenic than in prior years. Compared with Texas data for 2002–2011, we did not find any statistically significant differences in median ages of reported WNND or fatal cases in 2012 using the Kruskal-Wallis 1-way analysis of variance on ranks. Our findings from 2012 remain consistent with our experience from prior years; however, it remains critical to emphasize the importance of recognizing disease and testing persons of any age who have clinical signs and symptoms consistent with WNV infection.

The 2012 WNV outbreak in Texas greatly affected the state economically. On the basis of the acute medical care and productivity loss cost estimates provided by Barber et al. (*6*) (adjusted to 2012 USD), we crudely estimate the 2012 outbreak in Texas cost ≈$47.6 million (range $14.5–$140.7 million; Technical Appendix Table). In addition to these acute costs, the outbreak also required an increase in resources for mosquito control and public health efforts to respond to the epidemic. A recent study reported the cost of aerial spraying alone in Dallas County exceeded $1.6 million (*7*). The long-term economic impact of this outbreak also is expected to be substantial as a consequence of long-term rehabilitation and disability costs (*8*), possible risk for chronic kidney disease (*9*), and risk for premature death in severe cases (*10*).

The unprecedented 2012 outbreak confirms the need for continued vigilance for surveillance to enable timely implementation of control measures to prevent virus transmission. We expect Texas will continue to experience endemic levels of virus transmission with periodic epizootics. Considering the economic and physical costs to persons severely affected, development of an effective vaccine is urgently needed to prevent disease. Until a vaccine becomes available, public health authorities will need to maintain their focus on surveillance, disease recognition, implementation of control measures, and public education about protective measures.

Technical Appendix Calculations for economic costs of West Nile virus, Texas, USA, 2012.
