# Supplementary material for: West Nile Virus, Texas, USA, 2012
Source: Emerg Infect Dis. 2013 Nov;19(11):1836–8. doi: 10.3201/eid1911.130768 (PMC3837649; doi:10.3201/eid1911.130768)
Supplement: Technical Appendix — Calculations for economic costs of West Nile virus, Texas, USA, 2012. [file 13-0768-Techapp-s1.pdf]

Article DOI: http://dx.doi.org/10.3201/eid1911.130768

West Nile Virus, Texas, USA, 2012

Technical Appendix

Technical Appendix Table. Calculations for economic costs used for calculation of West Nile virus, Texas, USA, 2012*

Cost	Barber et al. cost estimate (*1*) (range)	Cost estimate adjusted to 2012 USD† (range)	No. cases	Estimated 2012 cost
WNND	$46,531 ($13,201–$140,257)	$54,702 ($15,519–$164,886)	844	$46,168,488 ($13,098,036–$139,163,784)
WNF	$1,170 ($1128–$1235)	$1,375 ($1326–$1452)	1,024	$1,408,000 ($1,357,824–$1,486,848)
Total				$47,576,488 ($14,455,860–$140,650,632)

*WNND, West Nile neuroinvasive disease; WNF, West Nile fever.
†Adjustment to 2012 US dollars made by using the Bureau of Labor Statistics Consumer Price Index Calculator (www.bls.gov/data/inflation_calculator.htm).

Reference

1. Barber LM, Schleier JJ III, Peterson RK. Economic cost analysis of West Nile virus outbreak, Sacramento County, California, USA, 2005. Emerg Infect Dis. 2010;16:480–6. PubMed http://dx.doi.org/10.3201/eid1603.090667
